# Effects of chromium-enriched bacillus subtilis KT260179 supplementation on chicken growth performance, plasma lipid parameters, tissue chromium levels, cecal bacterial composition and breast meat quality

**DOI:** 10.1186/s12944-016-0355-8

**Published:** 2016-11-08

**Authors:** Jiajun Yang, Kun Qian, Wei Zhang, Yayuan Xu, Yijing Wu

**Affiliations:** The Institute of Animal Husbandry and Veterinary Medicine, Anhui Academy of Agricultural Sciences, No. 40 Nongke South Road, Hefei, 230031 Anhui People’s Republic of China

**Keywords:** Chromium-enriched bacillus subtilis, Chicken, Growth performance, Meat quality, Bacterial composition

## Abstract

**Background:**

Both chromium (Cr) and probiotic *bacillus* own the virtues of regulating animal metabolism and meat quality. Purpose of this study was to evaluate the efficiency of supplemental Cr and *bacillus* in the form of chromium-enriched *Bacillus subtilis* KT260179 (CEBS) on chicken growth performance, plasma lipid parameters, tissue chromium levels, cecal bacterial composition and breast meat quality.

**Methods:**

Six hundred of 1-day-old Chinese Huainan Partridge chickens were divided into four groups randomly: Control, inorganic Cr, *Bacillus subtilis*, and CEBS. The feed duration was 56 days.

**Results:**

After 28 days of treatment, broiler feed CEBS or normal *B. subtilis* had higher body weights than control broiler, and after 56 days, chickens given either CEBS or *B. subtilis* had greater body weights than control broiler or those given inorganic Cr. Plasma total cholesterol, triglycerides, and low density lipoprotein cholesterol levels declined significantly in the CEBS group compared with the control, whereas plasma high density lipoprotein cholesterol levels increased significantly. The concentration of Cr in blood and breast muscle increased after CEBS and inorganic Cr supplementation. *B. subtilis* and CEBS supplementation caused a significant increase in the numbers of *Lactobacillus* and *Bifidobacterium* in the caecum, while the numbers of *Escherichia coli* and *Salmonella* decreased significantly compared to the control. Feed adding CEBS increased the lightness, redness, and yellowness of breast meat, improved the water-holding capacity, decreased the shear force and cooking loss.

**Conclusions:**

In all, CEBS supplementation promoted body growth, improved plasma lipid parameters, increased tissue Cr concentrations, altered cecal bacterial composition and improved breast meat quality.

## Background

With the application of modern breeding technology, the major biological characteristics of chickens or broilers, i.e. growth performance, daily weight gain, feed conversion efficiency and resistance to disease, have been improved significantly [1]. Despite the success of breeding programs in increasing meat production, the high selection intensity has resulted in negative impacts on growth performance and meat quality [2, 3].

Chromium (Cr) is an essential trace element and its beneficial effects on health are well documented in humans and animals [4]. Adding suitable dose of Cr to diet could improve the meat quality and regulate the metabolism of nutrient substances in animals [5]. Supplementation of the diet with trivalent Cr [Cr (III)] can be achieved using the salt chromium trichloride (CrCl_3_). Low molecular weight organic Cr complexes, such as picolinic acid and nicotinate salt forms, provide a myriad of benefits with higher organic bioavailability than the inorganic forms that are most often used as a dietary supplement [6]. Although Cr nanocomposites have even higher bioavailability than organic sources of Cr [7], their greater cost has inhibited widespread use. In animal husbandry, there is a need to explore a cheap and convenient organic source of Cr for use in industrial applications.

*Bacillus subtilis* is a probiotic bacterium that is widely used in diets of both humans and animals [8]. Oral administration of *B. subtilis* can exert a range of beneficial effects, including improvement of growth, enhancement of meat characteristics, optimizing the balance of intestinal microbiota, prevention and treatment of some diarrheal diseases, and reduction of serum cholesterol [9, 10]. For these reasons, *B. subtilis* has attracted considerable attention as a potentially beneficial dietary supplement for animal health.

It might be worthwhile to explore whether the combined use of organic Cr and *B. subtilis* might have a greater effect on regulating body growth and metabolism. Chinese Huainan Partridge chicken is a native breed in South of China. Because of special consumed habits, South of Chinese liked to choose the breed of broiler. Hence, Chinese Huainan Partridge chicken was employed to carry out this study. To test this hypothesis, we produce Cr-enriched *B. subtilis* (CEBS) which combines the virtues of *B. subtilis* and those of organic Cr and might induce an enhanced response to dietary supplementation. The aim of our study was to determine whether CEBS supplementation could play a role in improving body growth performance and breast meat quality, through improving lipid metabolism, utilization of Cr and intestinal bacterial composition on Chinese Huainan Partridge chicken.

## Methods

### Chicks, diets, and experimental design

The experimental protocol used in this study, including animal management, housing, and slaughter procedures, was approved by the Institution of Animal Science and Welfare of Anhui Province (Number: IASWAP2014110528). A total of 600 1-day-old Chinese Huainan Partridge chicken were randomly allocated into four groups with six replicate of 25 each. Chickens in the control group were fed basal feedstuff; the three treatments were fed basal feedstuff with different additives. Experimental diets were fed in two periods: starter (day 0–28) and finisher (day 29–56). The composition and nutrient analysis results for the basic diet are shown in Table 1. All the nutrients met or exceeded the nutrient requirements as recommended by the NRC [11]. The birds had free access to water and feed. In the whole trial, the normal immune procedure was implemented. *B. subtilis* KT260179 (submitted to National Center for Biotechnology Information) was provided by the Institute of Animal Husbandry and Veterinary Medicine, Anhui Academy of Agricultural Sciences in China. The fermentation of *B. subtilis* KT260179 and CEBS was followed Yang et al. (CEBS: Total Cr concentration 30 μg/g, Organic Cr 29.17 μg/g, Live *B. subtilis* KT260179 1.0 × 10^9^ CFU/g). After fermented, CEBS were harvested and added to mass feed followed 0.67 %. First, we added 0.67 L liquid CEBS to 9.33 kg mass feed and mixed with hand, then the mixed mass feed was added into blender contained 90 kg mass feed. The blender was employed for 20 min to mix the additives uniformity. The ways of *B. subtilis* KT260179 and inorganic Cr supplementation was the same as the CEBS. The ratio of *B. subtilis* KT260179 (Live *B. subtilis* KT260179 2.0 × 10^9^ CFU/g) supplemented was 0.34 %. We prepared the inorganic Cr solution and sterilized. Then, the CrCl_3_ solution was homogenize with mass feed. After prepared the four kinds of feedstuff, the population of *B. subtilis* was counted with plate method which yeast extract peptone dextrose medium was employed. Also, the concentration of Cr in four kinds of feedstuff was measured. The results were listed in Table 2.

**Table 1:** Composition and nutrient analysis of the basic diet for broilers at different stages (% as fed)

	Ingredient	Starter (0 ~ 28) %	Finisher (29 ~ 56) %
Item	Corn	57.97	61.75
	Soybean meal	29.30	26.45
	Fish powder	5.00	3.51
	Soybean oil	2.00	3.00
	Premix*	5.00^a^	5.00^a^
	Dicalcium phosphorus	0.47	0.29
	Limestone	0.26	0
Calculated nutrient	Metabolizable energy (MJ/kg)	12.12	12.54
	CP	21	19
	Calcium	1	0.9
	Total phosphorus	0.68	0.65
	Available phosphorus	0.45	0.38
	Lys	1.05	0.9
	Met	0.46	0.3

The premix provides

^a^vitamins and trace elements per kg diet: Vitamin A (retinyl acetate) 9, 875 IU, Vitamin D_3_ (cholecalciferol) 3, 000 IU, Vitamin E (DL-ɑ-tocopheryl acetate) 20 IU, menadione 3.25 mg, Vitamin B_12_ (cyanocobalamin) 0.025 mg, thiamin 1.5 mg, riboflavin 5.0 mg, biotin 0.032 mg, folacin 1.25 mg, niacin 12 mg, pantothenic acid 12 mg, and pyridoxine 3.75 mg, manganese 100 mg, zinc 80 mg, iron 80 mg, copper 8 mg, iodine 0.15 mg, and selenium 0.15 mg

**Table 2:** Concentration of Cr and number of *B. suthilis* in Groups

Groups	Concentration of Cr (ng Cr/g feed)	Number of *B. suthilis* (colony-forming units/g feed)
i (Control)	60.0	0
ii (Inorganic Cr)	260.0	0
iii (*B. suthilis*)	60.0	6.7 × 10^6^
iv (CEBS)	260.0	6.7 × 10^6^

### Growth performance and sample collections

Broilers in every replicate from each treatment group were weighed on day 0, 28, and 56. Daily feed consumption was accurately recorded. Daily weight gain and ratio of feed to gain (F/G) were calculated. ADG = body increase (g)/number of days. F/G = mass of food intake (g)/body increase (g).

After 56 days, two chickens from each replicate were selected, fasted for 12 h, and then tissues and blood were harvested under general halothane anesthesia. All blood samples were collected in 5.0 mL sterile heparinzed tubes. We removed 1 mL of each blood sample for measurement of Cr concentration. Then, remnant chick blood was centrifuged at 3000 g for 10 min to collect the plasma for biochemical assays (described below). Breast muscle was collected, which was frozen in liquid nitrogen and stored at 4 °C until analysis. The tissue of caeca from chickens was removed under aseptic conditions, which was stored in sterile plastic tubes in boxes packed with ice, and immediately sent to our laboratory for plate-counting of microorganisms.

### Plasma lipid analyses

Chicken plasma total cholesterol (TC), triglycerides (TG), low density lipoprotein cholesterol (LDLC), and high density lipoprotein cholesterol (HDLC) concentrations were measured using the appropriate detection kits (Nanjing Jiancheng Bioengineering Institute).

### Tissue Cr assay

A ZEEnit 700 P atomic absorption spectrometer (Analytik Jena, Germany) was employed for assaying chromium levels in tissues. All the measurements were performed by the method described by Afridi et al. [12]. Samples of blood, breast meat (0.2 g), and 0.2 g feedstuff from all four groups were placed in beakers and digested by addition of 10 mL of a nitric acid–perchloric acid (HNO3–HClO4) mixture. The mixture was heated on a sand bath until fumes appeared (the temperature was controlled at 200 °C by monitoring of the sand) and the solution had mostly evaporated. After cooling, 5 mL HNO_3_ was added, and the heating procedure was repeated at 180 °C. The cooled remainder was made up to 10 mL with distilled water. Eight replicates were used for each group.

### Cecal sample collection and bacterial composition analysis

The bacterial composition of ceaca in different treatments was done with plate method [13, 14] (eosin methylene blue agar for *Escherichia coli*, Salmonella-Shigellaagar plate for *Salmonella*, de Man, Rogosa, and Sharpe agar for total *Lactobacillus*, and BLB agar for *Bifidobacterium* by pour plate method, including *E. coli*, *Salmonella*, total *Lactobacillus*, and *Bifidobacterium* which were repeated three times).

### Meat Quality Analysis

Breast muscle samples were collected for meat quality at the end of the experiment. Lightness (L*), redness (a*), and yellowness (b*) of meat color was determined by a Chroma meter (model CR-410, Minolta Co., Tokyo, Japan). Water-holding capacity (WHC) was estimated by determining expressible juice using a modification of the filter paper press method described by Wierbicki and Deatherage [15] as follows. The breast meat were refrigerated overnight at 4 °C and then brought to room temperature before cooking to measure cooking loss and shear force. The breast meat from each bird was weighed and placed into a thin walled plastic bag, then cooked to an internal temperature of 70 °C on a digital thermostat water bath (HH-4, Jiangbo instrument, Jiangsu province, China) in a water-bath. Cooked meat was cooled to room temperature. The breast meat was weighed again for determination of cooking loss (%). Cutting three 1.9-mm-wide × 10 mm × 10 mm strips from the center of the muscles parallel to the muscle fibers through the thickest portion of the cooked muscle to measure meat shear force. The samples were cut perpendicular to the fiber direction using a Zwick Testing Machine Model Z2.5/TN1S (Zwick GmbH and Co, Germany) equipped with a Warner-Bratzler shear [16]. Peak force values were obtained in kg/mm^2^.

### Statistical analysis

Statistical analyses of the data were performed using SPSS 16.0 (SPSS, Inc., Chicago, IL, USA). Data are presented as means ± standard error (SE). Differences between groups were compared using analyses of variance. Differences between means were assessed by the Tukey’s honestly significant difference test of post hoc multiple comparisons. Data on Body weight, Average daily gain, Average daily feed intake and Ratio of feed to gain was statistically processed as REPEATED measurements. A *P* value of less than 0.05 was considered statistically significant.

## Results

### Growth performance

**Fig. 1:**
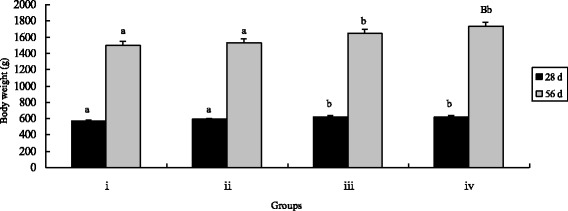
Effects of different treatments on chicken body weight. The chicken were treated with control (i), inorganic Cr (ii), *B. subtilis* (iii) and CEBS (iv) after 28 and 56 days. Bars represent mean ± S.E. Data of Body weight was statistically processed as REPEATED measurements. Bar in same color with different small letters a and b mean significant difference at 0.05 levels (*P* < 0.05). Aa, Bb mean significant difference at 0.01 levels (*P* < 0.01)

**Fig. 2:**
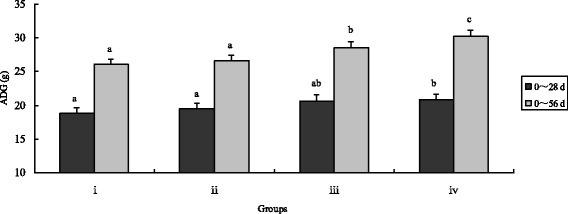
Effects of different treatments on chicken average daily gain. The chicken were treated with control (i), inorganic Cr (ii), *B. subtilis* (iii) and CEBS (iv) after 28 and 56 days. Bars represent mean ± S.E. Data of Body weight was statistically processed as REPEATED measurements. Bar in same color with different small letters a, b, c mean significant difference at 0.05 levels (*P* < 0.05)

**Fig. 3:**
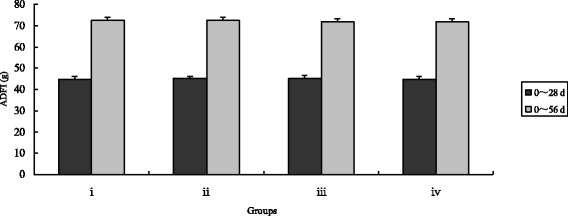
Effects of different treatments on chicken average daily feed intake. The chicken were treated with control (i), inorganic Cr (ii), *B. subtilis* (iii) and CEBS (iv) after 28 and 56 days. Bars represent mean ± S.E. Data of Body weight was statistically processed as REPEATED measurements. There were no differences among groups between 28 and 56 days (*P* > 0.05)

**Fig. 4:**
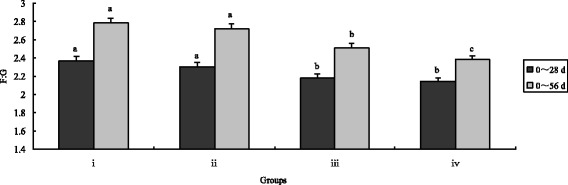
Effects of different treatments on chicken ratio of feed to gain. The chicken were treated with control (i), inorganic Cr (ii), *B. subtilis* (iii) and CEBS (iv) after 28 and 56 days. Bars represent mean ± S.E. Data of Body weight was statistically processed as REPEATED measurements. Bar in same color with different small letters a, b, c mean significant difference at 0.05 levels

Growth performance of Chinese Huainan Partridge chickens in different treatments was shown in Figs. 1, 2, 3 and 4. Chickens supplemented with *B. subtilis* KT260179 and CEBS owned higher final body weight than the control birds or those supplemented with inorganic Cr after 28 days (*P* < 0.05). The index of ADG among control, inorganic Cr and *B. subtilis* KT260179 supplemented groups was no significance (*P* > 0.05) at 28 days. While the result of F:G was opposite with the final body weight. Birds received *B. subtilis* KT260179 and CEBS had lower F:G (*P* < 0.05).

After 56 days, final body weights and ADG in CEBS group were significantly higher compared with the control and the inorganic Cr supplemented groups (*P* < 0.05) as well as the *B. subtilis* KT260179 group. While, the index of final body weight in group iv was highest of all (*P* < 0.05). Over the entire feeding duration, the F:G in the CEBS group was the lowest (*P* < 0.05). The F:G index of group iii was lower compared with the control and inorganic Cr groups (*P* < 0.05). There were no differences in the ADFI among the four groups either after 28 or after 56 days (*P* > 0.05).

### Plasma lipid parameters

The results of plasma lipid parameters were listed in Table 3. The levels of lipids (TC, TG, LDLC) in chickens given *B. subtilis* KT260179 were significantly lower compared with the controls, but were still higher than those of the CEBS group (*P* < 0.05). The concentrations of HDLC in the plasma of chicken in the CEBS and *B. subtilis* KT260179 groups were higher compared with the control chicken (*P* < 0.05), the former being higher in CEBS than *B. subtilis* KT260179 treatment (*P* < 0.05). The ratio of TC:LDLC was calculated, which indicated that chicken supplemented with CEBS had the highest ratio of all (*P* < 0.01). Chicken supplied with *B. subtilis* KT260179 showed higher TC:LDLC compared with the control and inorganic Cr groups (*P* < 0.01). The results for the ratio of TC:HDLC was that CEBS group was the lowest of all treatments (*P* < 0.01), and that for *B. subtilis* KT260179 was higher than that for CEBS but lower compared with inorganic Cr supplementation (*P* < 0.01). The result of TC:HDLC in control was highest of all (*P* < 0.01).

**Table 3:** Effects of different treatments on chicken breast meat characteristics

Groups	Meat color			WHC^4^ (%)	Shear force (kg/mm^2^)	Cooking loss (%)
	L^*1^	a^*2^	b^*3^			
i	51.85 ± 2.11^a^	12.63 ± 0.52^a^	16.14 ± 0.64^a^	66.62 ± 2.42^a^	2.79 ± 0.09^a^	16.89 ± 0.58^a^
ii	52.27 ± 2.09^a^	12.79 ± 0.50^a^	16.85 ± 0.62^a^	70.89 ± 2.39^a,b^	2.31 ± 0.08^b^	15.96 ± 0.57^a,b^
iii	54.08 ± 2.12^a^	13.14 ± 0.51^a^	16.92 ± 0.63^a^	71.99 ± 2.38^b^	2.24 ± 0.07^b^	15.01 ± 0.51^b^
iv	58.98 ± 2.08^b^	15.02 ± 0.49^b^	18.49 ± 0.58^b^	72.92 ± 2.40^b^	1.87 ± 0.10^c^	13.22 ± 0.48^c^

The different superscript small letters in the same column a, b mean significant difference at 0.05 levels (*P* < 0 · 05)

^1^Lightness

^2^Redness

^3^Yellowness

^4^Water-holding capacity

### Tissue chromium levels

**Fig. 5:**
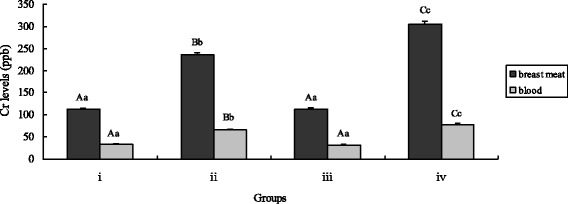
Effects of different treatments on chicken tissue chromium levels. The chicken were treated with control (i), inorganic Cr (ii), *B. subtilis* (iii) and CEBS (iv) after 28 and 56 days. Bars represent mean ± S.E. Data of Body weight was statistically processed as REPEATED measurements. Bar in same color with different small letters Aa, Bb mean significant difference at 0.01 levels (*P* < 0.01)

The Cr levels in breast meat and blood was measured with results shown in Fig. 5. The results indicated that chickens fed inorganic Cr had significantly more Cr in these tissues compared with the control and *B. subtilis* KT260179 supplementation groups (*P* < 0.01). Chickens in the CEBS group had the highest Cr levels in those two tissues (*P* < 0.01).

### Cecal bacterial composition

The cecal bacterial composition in the different groups of chickens was examined using the plate method (Table 4). Chicken given CEBS or *B. subtilis* KT260179 had lower numbers of *E. coli* and *Salmonella* compared with the control chicken (*P* < 0.05). There were no differences between the control and inorganic Cr-supplemented groups (*P* > 0.05). The numbers of *Lactobacillus* and *Bifidobacterium* in the CEBS and *B. subtilis* KT260179 groups increased significantly compared with the control and inorganic Cr-supplemented groups (*P* < 0.05).

**Table 4:** Effects of different treatments on cecal bacterial composition log_10_ CFU · g^−1^

Groups	Escherichia coli	Salmonella	Lactobacillus	Bifidobacterium
i	7.46 ± 0.14^a^	2.39 ± 0.03^a^	7.91 ± 0.15^a^	7.02 ± 0.06^a^
ii	7.42 ± 0.12^a^	2.33 ± 0.03^a^	7.93 ± 0.16^a^	7.05 ± 0.08^a^
iii	6.92 ± 0.13^b^	1.92 ± 0.04^b^	8.38 ± 0.16^b^	7.24 ± 0.06^b^
iv	6.88 ± 0.13^b^	1.88 ± 0.03^b^	8.37 ± 0.13^b^	7.26 ± 0.05^b^

The different superscript small letters in the same column a, b mean significant difference at 0.05 levels (*P* < 0 · 05)

### Breast meat quality

The breast meat quality on color L*, a*, b*, water-holding capacity, shear force and cooking loss were measured with 28-days-old broilers, and the results are provided in Table 5. Chickens with CEBS supplied had higher results of breast meat color L*, a* and b* (*P* < 0.05) than chickens in control, inorganic Cr and *B. subtilis* KT260179 groups (*P* < 0.05). Birds supplemented with CEBS and *B. subtilis* KT260179 had higher water-holding capacity and lower cooking loss than control group (*P* < 0.05). In further, the index of cooking loss in CEBS group was lowest of all (*P* < 0.05). The results of shear force in all three treatment groups were lower than that of control (*P* < 0.05). Also, the result of CEBS group was lowest of all (*P* < 0.05).

**Table 5:** Effects of different treatments on plasma lipid parameters

Groups	TC mmol/L	TG mmol/L	LDLC mmol/L	TC/LDLC	HDLC mmol/L	TC/HDLC
i	3.54 ± 0.11^a^	1.21 ± 0.05^a^	1.58 ± 0.06^a^	2.22 ± 0.06^Aa^	0.62 ± 0.03^a^	5.70 ± 0.13^Aa^
ii	3.47 ± 0.10^a^	1.15 ± 0.04^a^	1.51 ± 0.05^a^	2.29 ± 0.05^Aa^	0.65 ± 0.02^a^	5.33 ± 0.12^Bb^
iii	3.25 ± 0.12^b^	0.97 ± 0.05^b^	1.26 ± 0.04^b^	2.57 ± 0.04^Bb^	0.74 ± 0.03^b^	4.39 ± 0.14^Cc^
iv	3.03 ± 0.09^c^	0.82 ± 0.03^c^	1.16 ± 0.04^c^	2.61 ± 0.05^Cc^	0.92 ± 0.03^c^	3.29 ± 0.11^Dd^

*TC* total cholesterol, *TG* triglycerides, *LDLC* low density lipoprotein cholesterol, *HDLC* high density lipoprotein cholesterol, *TC/LDLC* ratio of TC to LDLC, *TC/HDLC* ratio of TC to HDLC

The different superscript small letters in the same column ^a, b, c^ mean significant difference at 0.05 levels (*P < 0 · 05*). ^Aa, Bb,^ ^Cc^ mean significant difference at 0.01 levels (*P < 0 · 01*)

## Discussion

Both of Cr and *B. subtilis* own the ability of modulating growth performance to chicken [14, 17, 18]. In our study, we supplemented a dose of 0.2 μg/g CrCl_3_ to basic feedstuff, which demonstrated that there was no promoting effect to Chinese Huainan Partridge chicken. Our results indicated that rates of growth were improved in chickens given *B. subtilis* KT260179 or CEBS supplements. Whereas chickens supplemented with CEBS had higher average body weights and greater feed utilization efficiency than controls. The F/G index of the CEBS group was the lowest over the entire feeding period, suggesting that this treatment was more efficient than *B. subtilis* alone in regulating body growth performance.

Dietary supplementary Cr and *B. subtilis* could improve body lipid metabolism [19–21]. Our data suggested that the concentration of plasma total cholesterol and triglycerides were decreased in chicken treated with CEBS or *B. subtilis*. CEBS enhanced the metabolism of TC and triglyceride. HDLC, synthesized mainly in the liver and the small intestine, plays an important part in eliminating serum cholesterol [22]. The decreased content of serum lipid resulted from a decrease of TG synthesis and/or enhancement of TG hydrolysis [23]. The decreased concentrations of hepatic TG revealed that lipid synthesis was reduced when the broilers were fed the *B. subtilis* supplemented diet, which can be associated with the improvement of plasma lipid metabolism. Likewise, supplementary inorganic Cr could improve the lipid metabolism to a slightly extent. Supplementation Cr in diet could modify the lipid metabolism especially in obesity or stress condition. The background of the supplementation of Cr in diabetic or fat persons is the tendency to lose the ability to convert Cr into a form that potentiates insulin action [24]. Therefore, nutritional supplements could decrease the lipid paraments in plasma [25]. For healthy animals, supplementary Cr would have little effect on lipid metabolism in a Cr-sufficient diet.

Previous studies have reported that Cr supplementation can increase the Cr content of tissues, although the results varied [26, 27]. In our study, the Cr contents of breast meat and blood were very significantly increased with the inorganic and CEBS supplemented. Our results here showed that the supplemental Cr in CEBS had a significant influence on Cr content of breast muscle in agreement with a previous report [28]. The effect of CEBS was greater than for inorganic Cr in this experiment, suggesting that CEBS had greater bioavailability as a Cr resource. The possible mechanism was that *B. subtilis* grew in medium contained inorganic Cr, to allow the efficient conversion of inorganic Cr into organic forms, such as amino acid chelated Cr, organic compounds are absorbed more efficiently than are inorganic forms Hence, CEBS owed more bioavailability than inorganic Cr [19, 29].

Probiotic supplements have been reported to modify the composition of the caecal microbiota [30, 31]. Similarly, our results here indicated that *B. subtilis* supplements could alter the bacterial flora in the caeca of treated chicken. Supplementation of 10^5^ to 10^9^ colony-forming units (CFU)/g of probiotics in the diet could exert beneficial effects in broilers [32]. However, Huang reported that a higher inclusion level did not always result in enhanced performance in poultry [33]. There were reports on supplemental doses 28, which suggested that 10^6^ CFU of Bacillus per gram feedstuff were suitable. In the present study, we supplemented 10^6^ CFU/g *B. subtilis*. The total Cr concentration in the CEBS treatment was 30 μg/g; we therefore added a 0.67 % supplement to the feedstuff, which conform the reported supplementary dose (0.2 μg Cr per gram feedstuff) [34, 35].

When probiotics are used for meat quality enhancement, the effects have been questioned, and many different results have been shown. Some authors reported advantages of probiotic supplementation [36, 37], whereas others reported no beneficial effects [38]. Meat quality could be modified by the improvement of Cr utilization and intestinal bacterial composition. Our result indicated that with *B. subtilis* supplemented there were no significances on meat color. While, dietary addition of CEBS could improve meat color, which advised that CEBS combined the virtue of Cr and *B. subtilis* regulating the nutrition metabolism strongly. In the measurement of meat quality, water-holding capacity, shear force and drip loss are important factors because some nutrients may be lost in the exudate by water loss, which may be reflected in the juiciness, tenderness, and flavor of meat [39]. In our results, index of water-holding capacity were significantly improved with *B. subtilis* and CEBS supplemented. Likewise, indexes of shear force and drip loss were significantly lowered. Those results suggested that *B. subtilis* supplied in feedstuff modified the breast meat quality. The effect of CEBS was greater than for CrCl_3_ and *B. subtilis* in the present experiment.

## Conclusions

The results of our study indicated that feeding supplementary CEBS combined the benefits of Cr and probiotics could improve chicken final body weight and ADG, decrease F:G, compared with control group. While there were no significances on ADFI. Chicken supplemented with CEBS could modify plasma lipid parameters, enhance tissue Cr concentrations and improve caecal bacterial composition and breast meat quality.

## Abbreviations

a*: Redness
ADG: Average daily weight gain
b*: Yellowness
*B. subtilis*: Bacillus subtilis
CEBS: Chromium enriched bacillus subtilis
Cr: Chromium
F/G: Ratio of feed to gain
HDLC: High density lipoprotein cholesterol
L*: Lightness
LDLC: Low density lipoprotein cholesterol
TC: Total cholesterol
TG: Triglycerides
WHC: Water-holding capacity

## References

[1] Havenstein G, Ferket P, Qureshi M (2003). Growth, livability, and feed conversion of 1957 versus 2001 broilers when fed representative 1957 and 2001 broiler diets. Poult Sci.

[2] Havenstein G, Ferket P, Qureshi M (2003). Carcass composition and yield of 1957 versus 2001 broilers when fed representative 1957 and 2001 broiler diets. Poult Sci.

[3] Le Bihan-Duval E, Millet N, Remignon H (1999). Broiler meat quality: effect of selection for increased carcass quality and estimates of genetic parameters. Poult Sci.

[4] Mertz W (1993). Chromium in human nutrition: a review. J Nutr.

[5] Lukaski HC (1999). Chromium as a supplement. Annu Rev Nutr.

[6] Laschinsky N, Kottwitz K, Freund B, Dresow B, Fischer R, Nielsen P (2012). Bioavailability of chromium (III)-supplements in rats and humans. Biometals.

[7] Eybe T, Audinot JN, Udelhoven T, Lentzen E, El Adib B, Ziebel J, Hoffmann L, Bohn T (2013). Determination of oral uptake and biodistribution of platinum and chromium by the garden snail (Helix aspersa) employing nano-secondary ion mass-spectrometry. Chemosphere.

[8] Khochamit N, Siripornadulsil S, Sukon P, Siripornadulsil W (2015). Antibacterial activity and genotypic-phenotypic characteristics of bacteriocin-producing Bacillus subtilis KKU213: potential as a probiotic strain. Microbiol Res.

[9] Kritas SK, Marubashi T, Filioussis G, Petridou E, Christodoulopoulos G, Burriel AR, Tzivara A, Theodoridis A, Piskorikova M (2015). Reproductive performance of sows was improved by administration of a sporing bacillary probiotic (Bacillus subtilis C-3102). J Anim Sci.

[10] Lei K, Li YL, Wang Y, Wen J, Wu HZ, Yu DY, Li WF (2015). Effect of dietary supplementation of Bacillus subtilis B10 on biochemical and molecular parameters in the serum and liver of high-fat diet-induced obese mice. J Zhejiang Univ Sci B.

[11] National Research Council (1994). Nutrient Requirement of Poultry.

[12] Afridi HI, Kazi TG, Talpur FN, Arain S, Arain SS, Kazi N, Panhwar AH, Brahman KD (2014). Evaluation of chromium and manganese in biological samples (scalp hair, blood and urine) of tuberculosis and diarrhea male human immunodeficiency virus patients. Clin Lab.

[13] Mountzouris KC, Tsirtsikos P, Kalamara E, Nitsch S, Schatzmayr G, Fegeros K (2007). Evaluation of the efficacy of a probiotic containing Lactobacillus, Bifidobacterium, Enterococcus, and Pediococcus strains in promoting broiler performance and modulating cecal microflora composition and metabolic activities. Poult Sci.

[14] Jeong JS, Kim IH (2014). Effect of Bacillus subtilis C-3102 spores as a probiotic feed supplement on growth performance, noxious gas emission, and intestinal microflora in broilers. Poult Sci.

[15] Wierbicki E, Deatherage FE (1958). Determination of water-holding capacity of fresh meats. Agric Food Chem.

[16] Cason JA, Lyon CE, Papa CM (1997). Effect of Muscle Opposition during Rigor on Development of Broiler Breast Meat Tenderness. Poult Sci.

[17] Jahanian R, Rasouli E (2015). Dietary chromium methionine supplementation could alleviate immunosuppressive effects of heat stress in broiler chicks. J Anim Sci.

[18] Lin YC, Huang JT, Li MZ, Cheng CY, Lien TF (2015). Effects of supplemental nanoparticle trivalent chromium on the nutrient utilization, growth performance and serum traits of broilers. J Anim Physiol Anim Nutr (Berl).

[19] Yang J, Xu Y, Qian K, Zhang W, Wu D, Wang C (2016). Effects of chromium-enriched Bacillus subtilis KT260179 supplementation on growth performance, caecal microbiology, tissue chromium level, insulin receptor expression and plasma biochemical profile of mice under heat stress. Br J Nutr.

[20] Sadeghi M, Najaf Panah MJ, Bakhtiarizadeh MR, Emami A (2015). Transcription analysis of genes involved in lipid metabolism reveals the role of chromium in reducing body fat in animal models. J Trace Elem Med Biol.

[21] Do HJ, Chung JH, Hwang JW, Kim OY, Lee JY, Shinl MJ (2015). 1-deoxynojirimycin isolated from Bacillus subtilis improves hepatic lipid metabolism and mitochondrial function in high-fat-fed mice. Food Chem Toxicol.

[22] Wang MQ, Xu ZR, Zha LY, Lindemann MD (2007). Effects of chromium nanocomposite supplementation on blood metabolites, endocrine parameters and immune traits in finishing pigs. Anim Feed Sci Technol.

[23] Walker SE, Register TC, Appt SE, Adams MR, Clarkson TB, Chen H, Isom S, Franke AA, Kaplan JR (2008). Plasma lipid-dependent and -independent effects of dietary soy protein and social status on atherogenesis in premenopausal monkeys: implications for postmenopausal atherosclerosis burden. Menopause.

[24] Wang ZQ, Zhang XH, Russell JC, Hulver M, Cefalu WT (2006). Chromium picolinate enhances skeletal muscle cellular insulin signaling in vivo in obese, insulin-resistant JCR:LA-cp rats. J Nutr.

[25] Toghyani M, Shivazad M, Gheisari A, Bahadoran R (2012). Chromium supplementation can alleviate the negative effects of heat stress on growth performance, carcass traits, and meat lipid oxidation of broiler chicks without any adverse impacts on blood constituents. Biol Trace Elem Res.

[26] Prescha A, Krzysik M, Zablocka-Slowinska K, Grajeta H (2014). Effects of exposure to dietary chromium on tissue mineral contents in rats fed diets with fiber. Biol Trace Elem Res.

[27] Uyanik F, Eren M, Guclu BK, Sahin N (2005). Effects of dietary chromium supplementation on performance, carcass traits, serum metabolites, and tissue chromium levels of Japanese quails. Biol Trace Elem Res.

[28] Zha LY, Xu ZR, Wang MQ, Gu LY (2007). Effects of chromium nanoparticle dosage on growth, body composition, serum hormones and tissue chromium in Sprague-Dawley rats. J Zhejiang Univ Sci B.

[29] Stewart RDH, Griffiths NM, Thomson CD, Robinson MF (1987). Quantitative selenium metabolism in normal New Zealand women. Br J Nutr.

[30] Salazar N, Binetti A, Gueimonde M, Alonso A, Garrido P, Gonzalez del Rey C, Gonzalez C, Ruas-Madiedo P, de los Reyes-Gavilan CG (2011). Safety and intestinal microbiota modulation by the exopolysaccharide-producing strains Bifidobacterium animalis IPLA R1 and Bifidobacterium longum IPLA E44 orally administered to Wistar rats. Int J Food Microbiol.

[31] Sen S, Ingale SL, Kim YW, Kim JS, Kim KH, Lohakare JD, Kim EK, Kim HS, Ryu MH, Kwon IK, Chae BJ (2012). Effect of supplementation of Bacillus subtilis LS 1–2 to broiler diets on growth performance, nutrient retention, caecal microbiology and small intestinal morphology. Res Vet Sci.

[32] Mountzouris KC, Tsitrsikos P, Palamidi I, Arvaniti A, Mohnl M, Schatzmayr G, Fegeros K (2010). Effects of probiotic inclusion levels in broiler nutrition on growth performance, nutrient digestibility, plasma immunoglobulins, and cecal microflora composition. Poult Sci.

[33] Huang MK, Choi YJ, Houde R, Lee JW, Lee B, Zhao X (2004). Effects of Lactobacilli and an acidophilic fungus on the production performance and immune responses in broiler chickens. Poult Sci.

[34] Anderson RA, Bryden NA, Polansky MM, Richard MP (1989). Chromium supplementation of turkeys: effects on tissue chromium. J Agric Food Chem.

[35] Hossain SM, Barreto SL, Silva CG (1998). Growth performance and carcass composition of broilers fed supplemental chromium from chromium yeast. Anim Feed Sci Technol.

[36] Mahajan P, Sahoo J, Panda PC (2000). Effect of probiotic (Lacto-Sacc) feeding, packaging methods and season on the microbial and organoleptic qualities of chicken meat balls during refrigerated storage. J Food Sci Technol.

[37] Zhou X, Wang Y, Gu Q, Li W (2010). Effect of dietary probiotic, Bacillus coagulans, on growth performance, chemical composition, and meat quality of Guangxi Yellow chicken. Poult Sci.

[38] Zhang ZF, Zhou TX, Ao X, Kim IH (2012). Effects of β-glucan and Bacillus subtilison growth performance, blood profiles, relative organ weight and meat quality in broilers fed maizesoybean meal based diets. Livest Sci.

[39] Chen H, Dong X, Yao Z, Xu B, Zhen S, Li C, Li X (2012). Effects of prechilling parameters on water-holding capacity of chilled pork and optimization of prechilling parameters using response surface methodology. J Anim Sci.

